# A putative, novel coli surface antigen 8B (CS8B) of enterotoxigenic *Escherichia* coli

**DOI:** 10.1093/femspd/ftv047

**Published:** 2015-07-17

**Authors:** Samuel M. Njoroge, Christine J. Boinett, Laure F. Madé, Tom T. Ouko, Eric M. Fèvre, Nicholas R. Thomson, Samuel Kariuki

**Affiliations:** 1 Centre for Microbiology Research, Kenya Medical Research Institute, PO Box 43640-00100 Nairobi, Kenya; 2 Wellcome Trust Sanger Institute, Hinxton, Cambridge CB10 1SA, UK; 3 Institute of Infection and Global Health, University of Liverpool, Liverpool L69 7BE, UK; 4 International Livestock Research Institute, PO Box 30709-00100, Nairobi, Kenya

**Keywords:** Enterotoxigenic *Escherichia coli*, type IVb pilins, coli surface antigen 8 subtype B, Kenya, colonization factors

## Abstract

Enterotoxigenic *Escherichia coli* (ETEC) strains harbor multiple fimbriae and pili to mediate host colonization, including the type IVb pilus, colonization factor antigen III (CFA/III). Not all colonization factors are well characterized or known in toxin positive ETEC isolates, which may have an impact identifying ETEC isolates based on molecular screening of these biomarkers. We describe a novel coli surface antigen (CS) 8 subtype B (CS8B), a family of CFA/III pilus, in a toxin producing ETEC isolate from a Kenyan collection. In highlighting the existence of this putative CS, we provide the sequence and specific primers, which can be used alongside other ETEC primers previously described.

## Abstract (precis)

The authors report on a new adhesin, that they have named coli surface antigen 8 subtype B (CS8B); to date, there are five variants of CS8 undetectable using current primers.

## Graphical abstract

**Graphical Abstract Figure.**
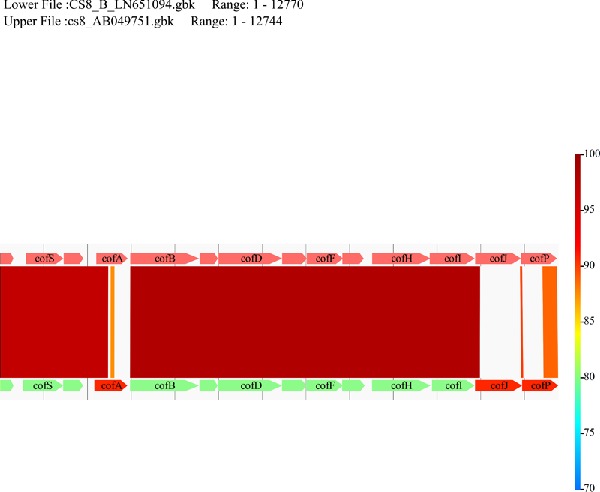
The authors report on a new adhesin, that they have named coli surface antigen 8 subtype B (CS8B); to date, there are five variants of CS8 undetectable using current primers.

Enterotoxigenic *Escherichia coli* (ETEC) pathotypes are a major cause of infantile watery diarrhea in developing countries as well as in travellers globally (Qadri *et al.* 2005). They are defined by the presence of a heat-labile toxin and/or a heat-stable toxin (Nataro and Kaper 1998). In addition, ETEC is known to carry an array of colonization factor antigens (CFAs) or adhesive fimbriae that promote adherence to and colonization of the host intestinal epithelial cells thus playing an important role in disease instigation and pathogenesis (Turner *et al.* 2006).

To date, 20 distinct colonization factors (CFs) have been identified in ETECs causing diarrhea in humans namely CFAI, CS1–8 and CS12–23 (Rodas *et al.* 2009; Mazariego-espinosa *et al.* 2010). ETECs may express one of two-type IVb pilli, CFA/III of coli surface antigen 8 (CS8) or Longus of coli surface antigen 21 (CS21). The CFA/III consists of a cluster of 14 member genes that make up the *cof* operon (Taniguchi *et al.* 2001; Yuen *et al.* 2013).

We describe a novel type IVb, CFA/III colonization factor designated CS8B, carried by an ETEC strain ESEI_111 obtained in September 2013 from a child under 5 years of age presenting with watery diarrhea. *Escherichia coli* strain ESEI_111 was sequenced at the Wellcome Trust Sanger Institute as part of a pilot global ETEC project. Genomic DNA was isolated by QIAamp® DNA mini Kit (Eigen, Courtaboeuf, France) and sequenced using the Illumina HiSeq2000 platform. Paired-end reads of 100 bp were assembled using *Velvet* (Zerbino 2011). For future epidemiological screening, primers for selected genes: *cof*J-B and *cof*P-B of CS8B were designed using SeqBuilder software (Table 1) and were tested on the ESEI_111 strain. All the PCR reactions (single or multiplex) were performed in 25 μl final volume containing 1 μl of the template DNA, 22 μl of water and 0.5 μl of each primer mixed with the Ready-To- Go PCR Beads (GE healthcare, Buckinghamshire, UK) according to manufacturer's specifications. The thermocycling conditions were as follows: 95°C for 2 min, 95°C for 30 s, 60°C for 30 s and 30 s at 72°C for 35 cycles, with a final 10 min extension at 72°C, and the PCRs were performed in the DNA Engine PTC-0221 Dyad Cycler. Multilocus sequence typing (MLST) was performed as previously described (Aanensen and Spratt 2005). Plasmid typing was carried out *in silico* using an in-house script and primer sequences described previously (Carattoli *et al.* 2005). Manual annotation was carried out using the genome viewer Artemis (Rutherford *et al.* 2000). Comparative genomics of CS8B to available complete CS8 operon coding sequences from European Nucleotide Archive (ENA) was performed using GenomeMatcher (Ohtsubo *et al.* 2008). To determine the phylogenetic relationship between nucleotide sequences of 33 *cofJ*, 17 *lngJ* and one *tcpJ*, a tree was inferred by using the maximum likelihood method based on the Tamura–Nei model using the poisson model of amino acid substitution, uniform rates among sites, with a 95% site coverage cut-off and 1000 bootstrap replicates (Tamura and Nei 1993). Percentage nucleotide identities were calculated using ClustalW Omega (Sievers *et al.* 2011). The KEMRI/National Ethics Review Committee SCC No 2507 approved all procedures.

**Table 1.** Primers designed for CS8B.

Primer name	5^′^ -3^′^ Sequence	Annealing Tm	Product size
CS8-JB_F	GCCGGGGATGGCATCACCA	64	968 bp
CS8-JB_R	GCGTTTATAAGAGGCTATCTG	60	
CS8-PB_F	CACTGGTATGTGTGTTGGTAGC	64	730 bp
CS8-PB_R	GGAAATTGCCGGGCCAAAAG	62	

ESEI_111, by MLST, belongs to sequence type 410, encodes a heat labile toxin (*eltA* and *eltB*) and harbors a multidrug-resistant IncI1 plasmid encoding *tetB*, *bla*_TEM-1_, *sul2*, *strA*, *ampC* and *dfrA8* resistance genes.

Although our understanding of the pathogenesis of ETEC has been accelerated by data from whole genome sequencing (WGS), not all CFs have been identified. Current moelcular screening of ETECs involves PCR amplification of the known toxin genes and CFs (Rodas *et al.* 2009). However, some ETECs are classed as CF negative either because they lack a CF or they may harbor unknown or divergent CFs. ESEI_111 is one such isolate lacking all known CFs, as defined by PCR, but haboring a CS8B operon. WGS of ESEI_111, followed by mapping of the resultant short reads to all known CFs using SMALT software version 0.6.4 and a 90% identity cut-off, showed that this isolate did in fact harbor a CF which was related, but distinct from the well-characterized CF CS8 operon. Figure 1 shows the comparative genomic analysis of *cofR*, *cofS*, *cofT*, *cofA*-*J* and *cofP* which are predicted to encode the CS8B CF protein with the orthologous cluster encoding CS8 (Taniguchi *et al.* 2001). These data showed that CS8B was a chimeric operon with *cofR*, *cofS*, *cofT*, *cofB*, *cofC*, *cofD*, *cofE*, *cofF*, *cofG*, *cofH* and *cofI* showing more than 96% sequence identity to their orthologs in the CS8 operon. However, *cofA*, *cofJ* and *cofP*, which encode the major subunit pilin, the secreted CF and the prepilin peptidase, respectively, were highly divergent showing less than 88% identity with their CS8 orthologs.

**Figure 1.**
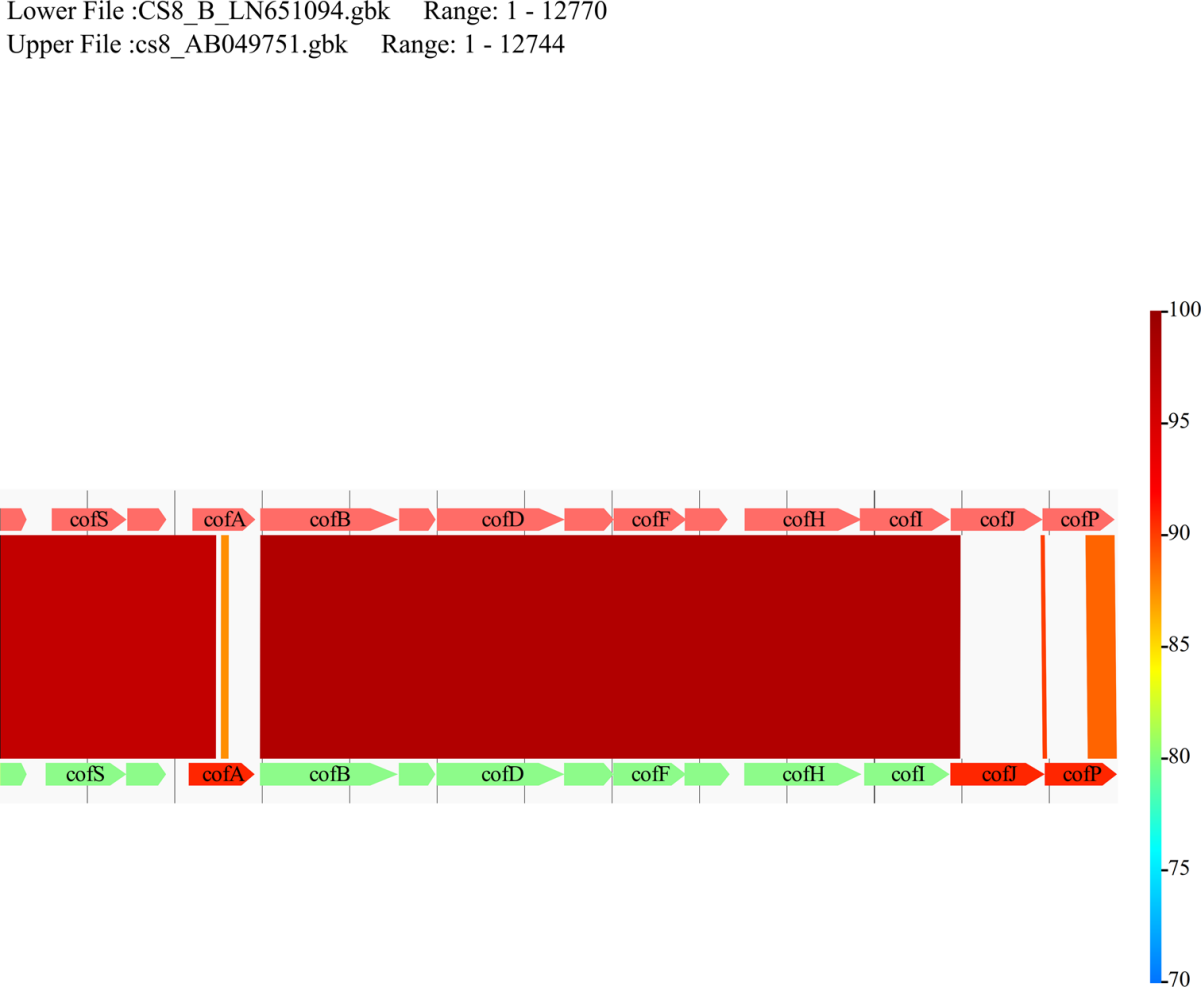
Comparative genomics for CS8 and CS8B from this study. Comparative genomics with CS8B *cof* operon from this study at the bottom and CS8 of accession AB049751 at the top using GenomeMatcher (Ohtsubo *et al*. 2008). *cofR*, *cofS*, *cofT*, *cofB*, *cofC*, *cofD*, *cofE*, *cofF*, *cofG*, *cofH* and *cofI* remain conserved with more than 96% similarity upstream. CFA/III gene products are *cofA*- major pilin, *cofB*- minor pilin, *cofD*- secretin, *cofE, cofF* and *cof*G- IMAPs (inner membrane accessory proteins), *cofH*- assembly ATPase, *cofI*- IMCP (inner membrane core protein), *cofJ*- secreted colonization factor, *cofP*- prepilin peptidase. Comparing CS8B *cof* operon to CS8 reveals divergence in *cofA*, *cofP* and *cofJ*, which encode the major subunit pilin, the prepilin peptidase and the secreted colonization factor, respectively. Colored scale on the right-hand side indicates percent nucleotide identity. Off-white areas between *cofA*, *cofJ* and *cofP* represent regions of no synteny.

Phylogenetic analysis of all known prepilin peptidases related to *cofJ* showed that CS8B, clusters distinctly from all other *cofJ* genes (Fig. 2), and homolog *tcpJ* of *Vibrio cholerae* (EAZ73994), which has 44% identity to the CS8B *cof*J gene identified in this study. We observed three distinct clusters of variant *cofJ* genes: Cluster I (1065 bp), Cluster II (1074 bp), III (1047 bp), IV (1122 bp). Cluster IIb share 99% nucleotide identity to *cofJ* described in this study, which we hypothesize to be CS8B. The *cofJ*s from this clade have 16 SNPs, resulting in 10 non-synonymous amino acid substitutions relative to accession CEJ09709 of CS8B. The CS8-JB primers described in this study amplify the *cofJ* of ESEI_111 (970 bp product) in addition to *cofJ* from Cluster IIb producing a 968 bp product by *in silico* PCR, and the five variants of CS8 (Cluster Ia/b, IIa/b, IIIb) currently undetectable using the current primers that amplify a region in the *cofA* gene.

**Figure 2.**
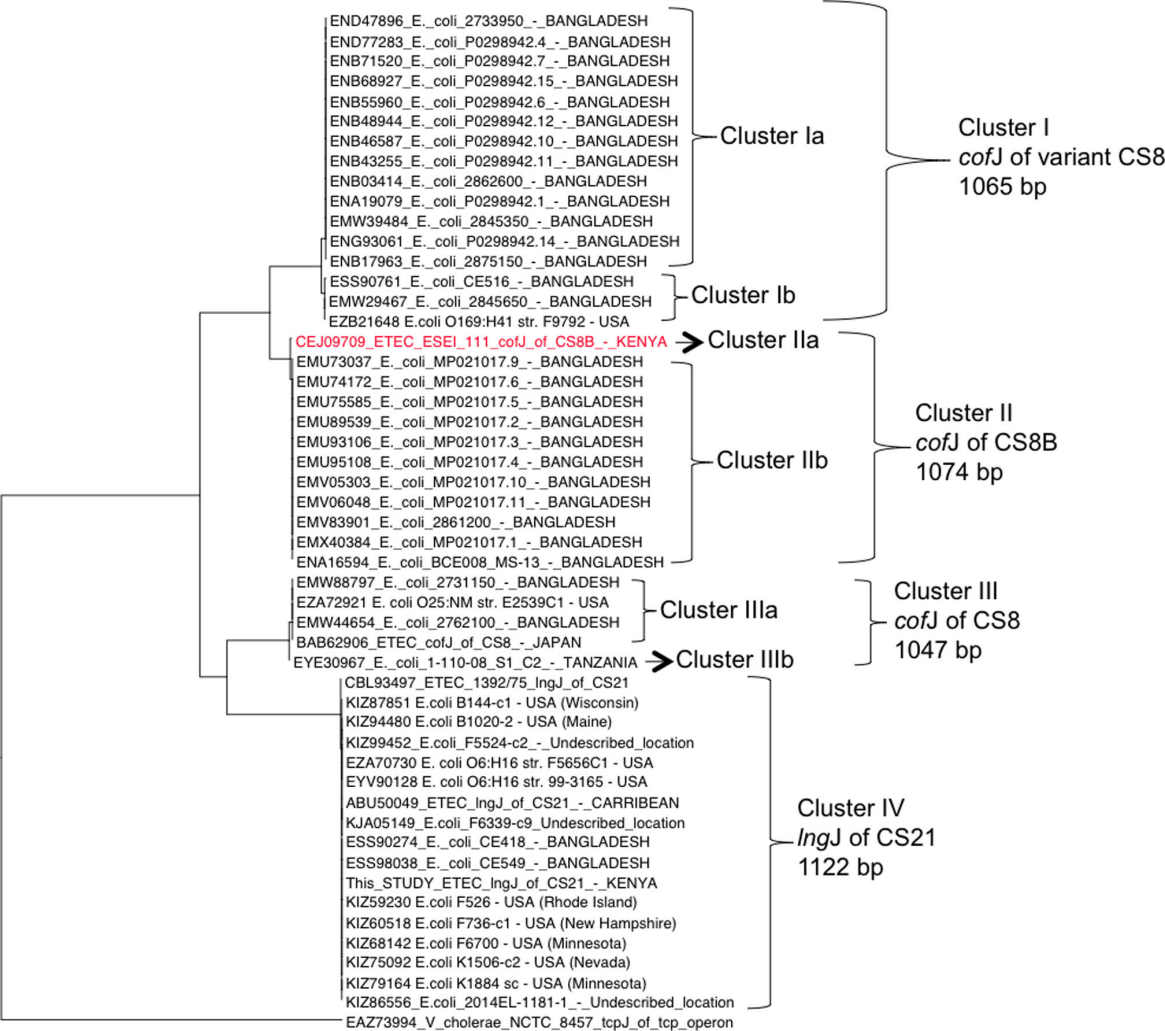
Phylogeny of CS8, CS8B from this study and CS21. Phylogenetic dendrogram constructed by the maximum likelihood method based on the Tamura–Nei model with MEGA 6.05, based on the complete major subunits of Type 4b pili gene sequences of *cofJ*, *tcpJ* and *lngJ* obtained from ENA. *cofJ* of CS8B from this study is shown in red. Most of the isolates originate from Bangladesh and those from other regions and their respective clusters are also indicated.

It is of note that unlike CS8, which is occasionally coexpressed with CS6 (Shaheen *et al.* 2003), CS8B was the only detected CF in the genome sequence of ETEC strain ESEI_111, novel or otherwise. Although other strains of CS8 variants were not available for *in vitro* testing, variants belonging to Cluster II can be covered by primers described in this study. Therefore, including CS8B primers that target *cofJ* and *cofP* alongside previously described primers (Rodas *et al.* 2009) when screening for CFs is recommended.

Considering that ETEC cause approximately 400 million diarrheal cases and almost 400 000 deaths per year in children under 5 years of age extending our ability to detect and monitor novel CFs found in low-/middle-income countries like Kenya will aid ETEC screening efforts and increase our ability to determine how the diversity of these genes may influence host–pathogen interactions. Importantly, this in turn may have future implications for vaccine development (Fleckenstein *et al.* 2010; Nada *et al.* 2011; Begum *et al.* 2014).

Although *cofJ* of CS8B have been detected before, we believe this is the first whole CS8B operon report, a variant of CS8. The annotated complete coding sequence reference for the CS8B *cof* operon is LN651094 and respective gene sequences have been deposited in the Genbank under accession numbers; *cofR*- CEJ09697, *cofS*- CEJ09698, *cofT*- CEJ09699, *cofA*- CEJ09700, *cofB*- CEJ09701, *cofC*- CEJ09702, *cofD*- CEJ09703, *cofE*- CEJ09704, *cofF*- CEJ09705, *cofG*- CEJ09706, *cofH*- CEJ09707, *cofI*- CEJ09708, *cofJ*- CEJ09709 and *cofP*- CEJ09710.
